# Optimal Divergence-Free Hatch Filter for GNSS Single-Frequency Measurement

**DOI:** 10.3390/s17030448

**Published:** 2017-02-24

**Authors:** Byungwoon Park, Cheolsoon Lim, Youngsun Yun, Euiho Kim, Changdon Kee

**Affiliations:** 1 School of Aerospace Engineering, Sejong University, Seoul 05006, Korea; byungwoon@sejong.ac.kr (B.P.); csleem@sju.ac.kr (C.L.); 2 KASS (Korean SBAS) Development Team/Satellite Navigation Team, Korea Aerospace Research Institute, Daejeon 34133, Korea; ysyun@kari.re.kr; 3 Mechanical and System Design Engineering Department, Hongik University, Seoul 04066, Korea; atomstan@gmail.com; 4 Institute of Advanced Aerospace Technology, School of Mechanical and Aerospace Engineering, Seoul National University, Seoul 08826, Korea

**Keywords:** GNSS, Hatch filter, divergence-free Hatch filter, smoothing window width, SBAS

## Abstract

The Hatch filter is a code-smoothing technique that uses the variation of the carrier phase. It can effectively reduce the noise of a pseudo-range with a very simple filter construction, but it occasionally causes an ionosphere-induced error for low-lying satellites. Herein, we propose an optimal single-frequency (SF) divergence-free Hatch filter that uses a satellite-based augmentation system (SBAS) message to reduce the ionospheric divergence and applies the optimal smoothing constant for its smoothing window width. According to the data-processing results, the overall performance of the proposed filter is comparable to that of the dual frequency (DF) divergence-free Hatch filter. Moreover, it can reduce the horizontal error of 57 cm to 37 cm and improve the vertical accuracy of the conventional Hatch filter by 25%. Considering that SF receivers dominate the global navigation satellite system (GNSS) market and that most of these receivers include the SBAS function, the filter suggested in this paper is of great value in that it can make the differential GPS (DGPS) performance of the low-cost SF receivers comparable to that of DF receivers.

## 1. Introduction

A global navigation satellite system (GNSS) provides two types of measurements, namely code and carrier-phase measurements. The measurement of the code observable is biased and coarse-ranged with a relatively large noise, while the carrier-phase measurement is indirect and ambiguous, but very precise [1]. The carrier phase is modulated by a chip sequence, which is composed of the data message modulated by the ranging code [2]. GNSS space vehicles transmit two or more carrier frequencies, including L1, the primary frequency, and L2, the secondary frequency [3,4]. The measurement errors are often categorized as noise or bias. A bias tends to persist over a period of time, which includes satellite-clock and ephemeris error, ionospheric delay, and tropospheric delay. Noise generally refers to a quickly varying error that averages out to zero over a short time interval [1].

To mitigate the bias error, differential GNSS (DGNSS) with the code or phase-range method is generally used, which improves the position accuracy with respect to the nearest base station [5,6]. The satellite-based augmentation system (SBAS) based on wide-area differential GPS (DGPS) is also an effective technique, which provides correction to users in the form of vectors parameterized by the error component on the L1 frequency [7].

High-end receivers requiring high accuracy to be used for land surveying or bridge monitoring typically receive multi-frequency signals to estimate ionospheric errors. On the other hand, low-cost GNSS receivers, which occupy most of the GPS market [8], generally contain the SBAS function to eliminate bias errors included in single-frequency (SF) measurements and use smoothing filters to reduce the effects of measurement noise remaining after bias removal. Park suggested the calculation of an optimal width using a noise model and the Klobuchar ionospheric model [9]. Zhang had applied the SBAS model [10] instead of the Klobuchar model to Park’s method, and Zhou proposed the use of the Doppler phase difference instead of the carrier phase difference when selecting the optimal window to prevent divergence [11]. Kim suggested the estimation of the ionospheric gradient to select the carrier-smoothing time [12].

Herein, we propose an SF Hatch filter that can compensate for the divergence due to ionospheric variation by the grid ionospheric vertical error (GIVE) from the SBAS message type (MT) 26. While the previous approaches only bound the ionospheric bias that is inevitably included in the estimates of the current Hatch filter; the proposed method reduces the divergence bias without any modification of the filter construction and properties. We focus on minimal modification and practical application of the classical Hatch filter, because the Hatch filter is a general filtering technique, which is used for code-based positioning after mitigating its errors by ground-based augmentation system (GBAS), SBAS, or national national DGPS (NDGPS).

The remainder of this paper is structured as follows: First, the existing smoothing filter and its error equations are briefly reviewed, and the review is repeated in the latter part of the paper. Second, the ionospheric-divergence mitigation technique with the SBAS message is discussed, following which the optimization algorithm is suggested. Subsequently, tests of real data are shown and analyzed. Lastly, discussion and conclusions are presented.

## 2. Overview of the Filter Using Carrier Phase Measurement

### 2.1. Overview of Smoothing Technology

A smoothing technique produces a time series in which spurious high-frequency noise is reduced. It is the most common method, mainly because it is the easiest way to understand and use. In spite of its simplicity, it is very effective to reduce random noise, as expressed in Equation (1) [13]:

(1) $$\mathrm{Typical}\;\mathrm{error}=\;\frac{\sigma }{\sqrt{N}}$$

where *N* is a finite number of samples, σ is the standard deviation of the Gaussian noise. The smoothing filter is optimal for reducing random noise, but it retains a sharp response, because of which it easily produces significant distortion with a large *N*.

To avoid large distortion, the moving average is generally used by averaging the same number (K) of neighboring samples (x). In equation form, the moving average (y) is expressed as follows:

(2) $$y\left[i\right]=\frac{1}{K}\overset{K-1}{\underset{j=0}{\sum }}x\left[i-j\right]$$

The moving-average filter is very common and is used for filtering GNSS pseudo-range measurements. GNSS measurements include high-dynamic terms, such as satellite-receiver range and clock bias, but most of them, except for ionospheric error, are common to the pseudo-range and carrier phase. If the ionospheric error of the carrier phase is corrected as that of the pseudo-range, as described in Equation (3), the pseudo-range of the k-epoch can be predicted without a serious bias by propagation using the carrier-phase variation and the observable from the i-epoch. This propagation process is expressed in Equation (4):

(3) Φ(k)= ϕ(k)+2I(k)

(4) $$\rho_{i}\left(k\right)=\;\rho \left(i\right)+\left\{\Phi \left(k\right)-\Phi \left(i\right)\right\}$$

where ρ(k) and ϕ(k) are the true pseudorange and carrier phase at the k-epoch, respectively. Here, the true value refers to the measurement, except for the user-side error, multi-path, and noise. I(k) is the ionospheric error in the pseudorange, Φ(k) is the iono-corrected carrier phase, and $\rho_{i}\left(k\right)$ is the pseudo-range prediction at the k-epoch, which propagated from the i-epoch. 

The measurement and propagated value from the observables include the multi-path and noise; thus, the measured pseudo-range at the k-epoch and the propagated pseudo-range from the i-epoch measurement are expressed by Equations (5) and (6), respectively:

(5) $$\rho_{k}\left(k\right)=\;\rho \left(k\right)+\upsilon_{\rho }\left(k\right)$$

(6) $$\rho_{i}\left(k\right)=\;\rho \left(k\right)+\left\{\upsilon_{\phi }\left(k\right)-\upsilon_{\phi }\left(i\right)\right\}+\upsilon_{\rho }\left(i\right)$$

where $\upsilon_{\rho }$ and $\upsilon_{\phi }$ are the user-side error in the pseudo-range and carrier phase, respectively.

The moving average of $\rho_{i}\left(k\right)$, the smoothing window of which is K, is expressed as follows:

(7) $$\hat{\rho }\left(k\right)\equiv \;\frac{1}{K}\overset{k}{\underset{i=k-K+1}{\sum }}\rho_{i}\left(k\right)$$

Assuming that $\upsilon_{\rho }$ and $\upsilon_{\phi }$ are Gaussian with zero mean and that $\upsilon_{\phi }$ is far smaller than $\upsilon_{\rho }$, the standard deviation of the smoothed pseudorange ($\hat{\rho }\left(k\right)$), $\sigma_{\hat{\rho }\left(k\right)}$, is almost $\frac{\sigma }{\sqrt{K}}$, as expressed by Equation (8):

(8) $$\sigma_{\hat{\rho }\left(k\right)}\approx \frac{\sigma }{\sqrt{K}}$$

### 2.2. Hatch Filter 

In a typical smoothing technique, the noise decreases as the measured value increases, and the Hatch filter expressed by Equation (9), which is a kind of smoothing filter, has the same characteristics. It is a type of recursive filter that uses the current measurement and previous estimate [14] without any dynamic model or additional sensors, and it can be implemented for a real-time operation in a low-cost SF GNSS receiver.

(9) $${\hat{\rho }}_{Hatch}\left(k\right)=\frac{K-1}{K}\left\{{\hat{\rho }}_{Hatch}\left(k-1\right)+\Delta \phi \left(k\right)\right\}+\frac{1}{K}\rho_{k}\left(k\right)$$

Δϕ(k)≡ϕ(k)−ϕ(k−1)

A discontinuity in the phase measurement is a significant threat to the Equation (4), which is an important basis of the Hatch filter. Thus, we should consider a cycle-slip detection when constructing the filter. Forming the code and phase combination (ρ−ϕ) cancels the non-dispersive delay such as geometry, clocks, and troposphere, and the remaining variables in the combination represent the phase ambiguity, instrumental delays, ionospheric refraction, and measurement noise. To monitor the cycle-slip using the time-difference of the code-minus-carrier, the ionospheric variation and measurement noise level should be considered. The ionospheric term varies slowly with time, far less than 2 cm/s [15] and, thus, only a small fraction of the cycle changes between consecutive epochs due to the ionosphere. On the other hand, the original code measurement noise may reach several cycles and may need to be smoothed. Since the code noise of the Hatch filter is already smoothed, the filtered pseudo-range and carrier phase combination (${\hat{\rho }}_{Hatch}-\phi$) is a good indicator for the cycle-slip detector. Once the cycle slip is detected, the filter is reset without any repair because the performance of the single frequency detector is not as good as that of the dual frequency.

Even if the cycle-slip does not occur, a bias error inevitably occurs as the averaging constant increases because the change in the pseudo-range is not exactly the same as the change in the carrier phase. The propagated pseudo-range of the Hatch filter from the measurement before the j-epoch, $\rho_{\;Hatch,k-j+1}\left(k\right)$, is expressed as follows [9]:

(10) $$\rho_{\;Hatch,k-j+1}\left(k\right)=\;\rho \left(k-j+1\right)+\left\{\phi \left(k\right)-\phi \left(k-j+1\right)\right\}+\upsilon_{\rho }\left(k-j+1\right)$$

Unlike Equation (4), Equation (10) uses a pure carrier phase, rather than an iono-corrected carrier phase. Thus, the error equation of $\rho_{\;Hatch,k-j+1}\left(k\right)$ is:

(11) $$\rho_{\;Hatch,k-j+1}\left(k\right)=\rho \left(k\right)-\left\{2\dot{I}\cdot j\right\}+\{\upsilon_{\phi }\left(k\right)-\upsilon_{\phi }\left(k-j+1\right)+\upsilon_{\rho }\left(k-j+1\right)\}$$

From Equation (11), a bias component, $\left\{2\dot{I}\cdot j\right\}$, increases as measurements for the filter are accumulated, while a noise component, $\left\{\upsilon_{\phi }\left(k\right)-\upsilon_{\phi }\left(k-j+1\right)+\upsilon_{\rho }\left(k-j+1\right)\right\}$, decreases. $\dot{I}$ is the temporal gradient of the ionospheric delays in the code and carrier measurements. Equation (11) indicates that the gradient is the only type of temporal correlation that would induce an undesired bias in the filtered measurements at the cost of noise reduction. Considering the size of $\upsilon_{\phi }$ is negligibly small compared to that of $\upsilon_{\rho }$ [1], the root mean square (RMS) error of the smoothed pseudo-range by the Hatch filter, the smoothing window width of which is K, can be described as follows [9]:

(12) $$RMS^{2}=\frac{1}{K}\sigma_{\rho \left(k\right)}^{2}+4{\left(K-1\right)}^{2}{\left(1-\frac{1}{e}\right)}^{2}{\dot{I}}^{2}$$

where $\upsilon_{\rho }\left(k\right)\sim N\left(0,\;\sigma_{\rho \left(k\right)}^{2}\right)$.

### 2.3. Divergence-Free Hatch Filter for GNSS Dual-Frequency Measurement

Although the Hatch filter offers excellent noise reduction and ease of implementation under normal conditions, the filter amplifies ionosphere errors under rare, anomalous storm conditions or with a large smoothing window width. To overcome this problem, a divergence-free Hatch filter was introduced. Recalling Equation (4), the ionosphere-related term ($-\left\{2\dot{I}\cdot j\right\}$) in the propagated measurement of the Hatch filter in Equation (11) should be compensated by the estimation of ionospheric delay, as described in Equation (13):

(13) $$\rho_{DF-divfree,k-j+1}\left(k\right)=\rho_{k-j+1}\left(k-1\right)+\left[\Delta \phi \left(k\right)+2\Delta \hat{I}\left(k\right)\right]+\upsilon_{\rho }\left(k-j+1\right)$$

where Δ is the time difference and $\hat{I}$. is the estimation of the ionospheric delay.

The ionospheric delay can be estimated using the dual-frequency (DF) GNSS observable, and the time difference of $\hat{I}$. is calculated using Equation (14) under the condition that no cycle slip occurs:

(14) $$\Delta {\hat{I}}_{DF}\left(k\right)=\frac{\Delta \phi_{L1}\left(k\right)-\Delta \phi_{L2}\left(k\right)}{\gamma -1}$$

where ${\hat{I}}_{DF}$ is the ionospheric delay estimation from the DF measurement; $\phi_{L1}$ and $\phi_{L2}$ are carrier-phase measurements for the frequencies L1 and L2, respectively; and γ is the squared ratio of the frequencies L1 and L2, $\frac{f_{1}^{2}}{f_{2}^{2}}$.

Equation (10) is corrected using ${\hat{I}}_{DF}$ as follows:

(15) $$\rho_{DF-divfree,k-j+1}\left(k\right)=\rho_{k-j+1}\left(k-1\right)+\left[\Delta \phi_{L1}\left(k\right)+2\Delta {\hat{I}}_{DF}\left(k\right)\right]+\upsilon_{\rho }\left(k-j+1\right)$$

$\rho_{DF-divfree,k-j+1}$ includes only noise error, $\upsilon_{\rho }\left(k-j+1\right)$, and does not contain a serious bias error, as shown in Equation (16). Therefore, the more the number of measurements used for the DF divergence-free Hatch filter of Equation (17), the better is the theoretical performance. However, this technique can be implemented only in DF receivers, and several papers have introduced new methods to mitigate the errors for low-cost SF receivers.

(16) $$\rho_{DF-divfree,k-j+1}\left(k\right)=\rho \left(k\right)+\upsilon_{\rho }\left(k-j+1\right)$$

(17) $${\hat{\rho }}_{DF-divfree}\left(k\right)=\frac{K-1}{K}\left[{\hat{\rho }}_{DF-divfree}\left(k-1\right)+\left\{\Delta \phi_{L1}\left(k\right)+2\Delta {\hat{I}}_{DF}\left(k\right)\right\}\right]+\frac{1}{K}\rho_{k}\left(k\right)$$

## 3. Divergence-Free Hatch Filter Algorithm for GNSS Single-Frequency Measurement

### 3.1. Divergence Reduction Using Ionospheric Correction of SBAS 

As described previously, it is difficult to estimate the ionospheric-error by SF measurement for a single epoch, and the recently proposed methods focus on bounding the divergence due to the ionospheric error, rather than on reducing it. The present paper proposes the ionospheric correction of SBAS to mitigate the ionospheric divergence of the conventional Hatch filter, as described in Equation (18):

(18) $$\rho_{SF-divfree,k-j+1}\left(k\right)=\rho_{k-j+1}\left(k-1\right)+\left[\Delta \phi \left(k\right)+2\Delta I_{s}\left(k\right)\right]+\upsilon_{\rho }\left(k-j+1\right)$$

Most of the low-cost SF GNSS receivers include the SBAS function, which provides the ionosphere-related term in the correction message. Therefore, the SF divergence-free Hatch filter suggested in this paper is plausible, even though its estimation performance of the ionospheric error is not as good as that of the DF divergence-free Hatch filter.

**Figure 1:**
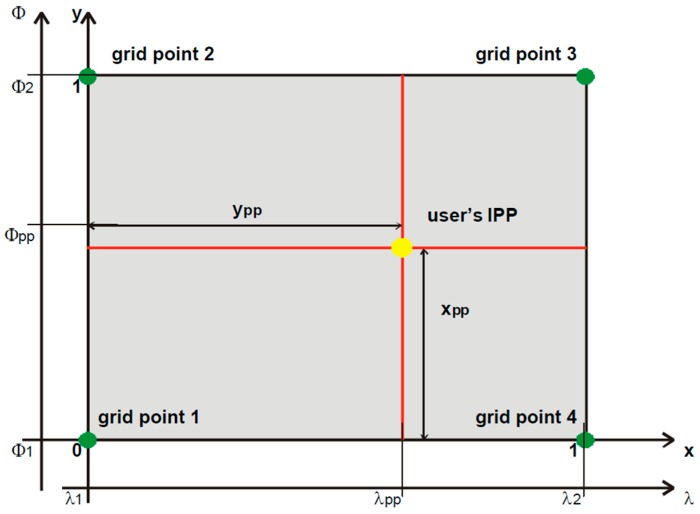
Interpolation concept of the ionospheric vertical delay.

The MT 26 ionospheric delay correction message of the SBAS provides the users with vertical delay (relative to an L1 signal) at geographically defined ionospheric grid points (IGPs). The SBAS user needs to interpolate from the broadcast IGP delays to calculate the ionospheric vertical delay at its computed ionospheric pierce point (IPP), as shown in Figure 1. 

Once the user establishes the vertical delay $I_{v}$ at the IPP, the user can multiply it by the obliquity factor $F_{pp}\left(El\right)$ [16] to obtain the ionospheric slant delay $I_{s}$:

(19) $$I_{s}\left(k\right)=I_{v}\left(k\right)\cdot F_{pp}\left(El\right)$$

(20) $$F_{pp}\left(El\right)={\left[1-{\left(\frac{R_{e}\cos\left(El\right)}{R_{e}+h}\right)}^{2}\right]}^{-\frac{1}{2}}$$

where $R_{e}$ and h are the approximate radius of Earth (6378.1363 km) and the height of the maximum electron density (350 km), respectively, and El is the elevation angle of the satellite from the user’s location.

Therefore, the SF divergence-free Hatch filter is proposed as follows:

(21) $${\hat{\rho }}_{SF-divfree}\left(k\right)=\frac{K-1}{K}\left[{\hat{\rho }}_{SF-divfree}\left(k-1\right)+\left\{\Delta \phi \left(k\right)+2\Delta I_{s}\left(k\right)\right\}\right]+\frac{1}{K}\rho_{k}\left(k\right)$$

### 3.2. Error Analysis of the SF Divergence-Free Hatch Filter 

Even though we assume that the SBAS has modelled current ionospheric delay sufficiently to ignore the bias error between I and $I_{s}$, it is necessary to consider the residual error due to the coarse resolution ($q_{I_{v}}$) of the IGP vertical delay, 0.125 m.

(22) $$\rho_{SF-divfree,k-j+1}\left(k\right)=\rho \left(k\right)+\delta \Delta I_{k-j+1}\left(k\right)+\upsilon_{\rho }\left(k-j+1\right)$$

where $\delta \Delta I\left(k\right)\equiv \Delta I\left(k\right)-\Delta I_{s}\left(k\right)$.

δΔI(k) is modelled by Equations (23) and (24), and $\delta {\dot{I}}_{v}$ is regard as 0 because $I_{v}$ is constant until a new MT 26 is updated.

(23) $$\delta \Delta I\left(k\right)=\Delta \delta I_{s}\left(k\right)\approx \delta {\dot{I}}_{s}\left(k\right)\cdot \Delta t_{k}$$

(24) $$\delta {\dot{I}}_{s}\left(k\right)=\delta {\dot{I}}_{v}\left(k\right)\cdot F_{pp}\left(El\right)+\delta I_{v}\left(k\right)\cdot {\dot{F}}_{pp}\left(El\right)$$

(25) $$\delta {\dot{I}}_{s}\left(k\right)\approx \delta I_{v}\left(k\right)\cdot {\dot{F}}_{pp}\left(El\right)$$

${\dot{F}}_{pp}\left(El\right)$ is obtained by differentiating Equation (25) as follows:

(26) $${\dot{F}}_{pp}\left(El\right)=-{\left[1-{\left(\frac{R_{e}\cos\left(El\right)}{R_{e}+h}\right)}^{2}\right]}^{-\frac{3}{2}}{\left(\frac{R_{e}}{R_{e}+h}\right)}^{2}\cos\left(El\right)\sin\left(El\right)\dot{El}$$

Assume that $\delta {\dot{I}}_{s}\left(k\right)$ is not changed during the last j-epoch, since $I_{s}\left(k\right)$ slowly changes (<0.02 m/s) [15] so that the quantization error can be regarded as the same for a while.

(27) $$\delta \Delta I_{s,k-j+1}\left(k\right)=\delta {\dot{I}}_{s}\left(k\right)\cdot j\cdot \Delta_{t}$$

where $\Delta_{t}$ is the sampling time and is set as 1 s in this paper.

Thus, the error equations of the propagated value of $\rho_{SF-divfree,k-j+1}\left(k\right)$ are expressed as Equations (28) and (29):

(28) $$\delta \rho_{SF-divfree,k-j+1}\left(k\right)=\delta {\dot{I}}_{s}\left(k\right)\cdot j+\upsilon_{\rho }\left(k-j+1\right)$$

(29) $$\delta \rho_{SF-divfree,k-j+1}\left(k\right)=\delta I_{v}\left(k\right)\cdot {\dot{F}}_{pp}\left(El\right)\cdot j\;+\upsilon_{\rho }\left(k-j+1\right)$$

### 3.3. Optimization Algorithm of the SF Divergence-Free Hatch Filter Performance

Equation (21) is equivalent to Equation (30); thus, the error of the estimation is expressed as Equation (31):

(30) $${\hat{\rho }}_{SF-divfree}\left(k\right)=\frac{1}{K}\overset{K}{\underset{j=1}{\sum }}\left\{\rho \left(k\right)+\delta {\dot{I}}_{s}\left(k\right)\cdot j+\upsilon_{\rho }\left(k-j+1\right)\right\}$$

(31) $$\delta {\hat{\rho }}_{SF-divfree}\equiv {\hat{\rho }}_{SF-divfree}\left(k\right)-\rho \left(k\right)=\frac{1}{K}\overset{K}{\underset{j=1}{\sum }}\delta {\dot{I}}_{s}\left(k\right)\cdot j+\frac{1}{K}\overset{K}{\underset{j=1}{\sum }}\upsilon_{\rho }\left(k-j+1\right)$$

Therefore, the variance (*V*) of the estimation error is obtained as Equation (32) from Equations (29) and (31), assuming that both $\delta {\dot{I}}_{s}$ and $\upsilon_{\rho }$ are zero-mean, independent, and identically distributed:

(32) $$V\left\{\delta {\hat{\rho }}_{SF-divfree}\right\}=\;\frac{1}{K^{2}}V\left\{\overset{K}{\underset{j=1}{\sum }}\delta I_{v}\left(k\right)\cdot {\dot{F}}_{pp}\left(El\right)\cdot j\right\}+\frac{1}{K^{2}}V\left\{\overset{K}{\underset{j=1}{\sum }}\upsilon_{\rho }\left(k-j+1\right)\right\}$$

$\delta I_{v}\left(k\right)$ is a quantization error, the variance of which is $\frac{q_{I_{v}}}{12}$ [17], and the variance of $\upsilon_{\rho }$ is $\sigma_{\rho \left(k\right)}^{2}$. Suppose that ${\dot{F}}_{pp}\left(El\right)$ and $\sigma_{\rho \left(k\right)}^{2}$ are constant during the recent k-epochs; then, Equation (32) is simplified as follows:

(33) $$V\left\{\delta {\hat{\rho }}_{SF-divfree}\right\}=\;\frac{{\dot{F}}_{pp}\left(El\right)\cdot q_{I_{v}}}{36}K+\left(\frac{{\dot{F}}_{pp}\left(El\right)\cdot q_{I_{v}}}{72}+\sigma_{\rho \left(k\right)}^{2}\right)\frac{1}{K}+\frac{q_{I_{v}}\cdot {\dot{F}}_{pp}\left(El\right)}{24}$$

Equation (33) is minimized when:

(34) $$\frac{{\dot{F}}_{pp}\left(El\right)\cdot q_{I_{v}}}{36}K=\left(\frac{{\dot{F}}_{pp}\left(El\right)\cdot q_{I_{v}}}{72}+\sigma_{\rho \left(k\right)}^{2}\right)\frac{1}{K}$$

Therefore, the performance of the SF divergence-free Hatch filter is optimized when:

(35) $$K=round\left(\sqrt{\frac{1}{2}+\frac{36\sigma_{\rho \left(k\right)}^{2}}{{\dot{F}}_{pp}\left(El\right)\cdot q_{I_{v}}}}\right)$$

## 4. Performance of the SF Divergence-Free Hatch Filter

### 4.1. Configuration of Field Test

To verify the suggested SF divergence-free Hatch filter algorithm and to evaluate its performance, we applied wide-area augmentation system (WAAS) correction to the Universal NAVSTAR Consortium (UNAVCO) P057 site, which is located in Snowville, UT, USA. Twenty-four hour observable data and navigation data logged on the day of year (DOY) 265 at 1-s intervals were obtained in the format of receiver independent exchange (RINEX), and the SBAS message from the pseudo-random noise (PRN) 135 GEO satellite was selected for data processing. 

**Figure 2:**
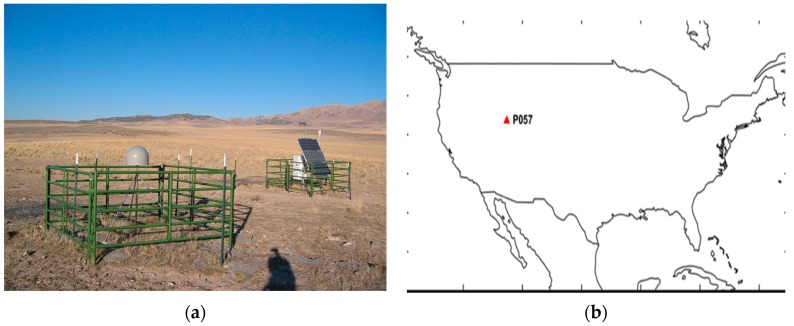
P057 site (**a**) overview of the site; (**b**) location of the site.

Since a NetRS receiver (Trimble, Sunnyvale, CA, USA) which provides DF observables, is implemented in the P057 reference station, the DF divergence-free Hatch filter can be applied to the measurement, as well as the conventional Hatch filter and SF divergence-free Hatch filter. It is expected that there will be no serious multi-path error, since there is no obstacle near the station, as shown in Figure 2.

### 4.2. Algorithm Development and Verification

Before implementing the suggested algorithm to the real data, we need to develop it specifically and verify its important assumption. In the SF divergence-free Hatch filter expressed by Equations (21) and (35), the standard-deviation function of the code noise and multipath for the elevation angle, $\sigma_{\rho }\left(El\right)$, is not yet defined. We also need to verify the important assumption that the ionospheric variation is estimated by the SBAS MT26 as well as by the linear combination of the L1/L2 carrier phase. 

#### 4.2.1. Pseudo-range Noise and Multi-Path Model Estimation

The measurement noise and multi-path depend on the elevation angle, resulting primarily from the antenna gain pattern and signal attenuation due to atmospheric effects. Lower satellites are subject to substantially higher noise; however, different hardware might produce different noise, depending on the elevation angle.

In this paper, we used the receiver model introduced by Kee [18] for this algorithm, as expressed by Equation (36):

(36) $$\sigma_{\rho }\left(El\right)=x_{0}+x_{1}\cdot e^{\left(-\frac{El}{x_{2}}\right)}$$

To fit the real code noise and multi-path (CNMP) to the model described in (36), the CNMP errors included in the code measurement are calculated by the difference between the leveled iono-corrected carrier phase after a post-processing and the pseudorange. Then, the parameters $x_{0},x_{1},x_{2}$ of Equation (36) are estimated using the fminsearch function of MATLAB as listed in Table 1.

**Figure 3:**
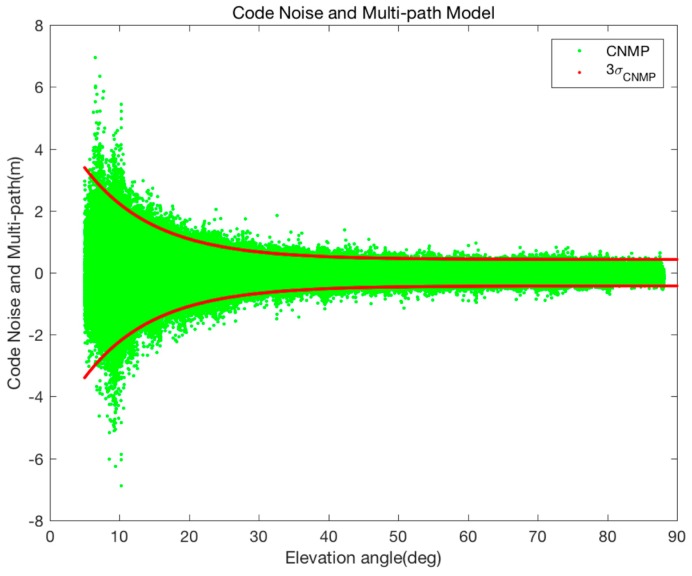
Code noise and multi-path modeling result.

Figure 3 shows the CNMP distribution for the elevation angle and the 3-sigma line estimated by the model from Equation (36) and Table 1. The figure shows that the number of samples exceeding the 3-sigma line is 6942 among 764,429 data; that is, the red line bounds 99.09% of the CNMP error. This means that the model described above reflects the Gaussian property of CNMP.

#### 4.2.2. Temporal Correlation Property of the Ionospheric Error

As shown in Equation (12), the performance of the typical Hatch filter depends on the noise size of the code measurement and the temporal correlation of the ionospheric error. Equation (18) indicates that the suggested Optimal SF divergence-free Hatch filter adjusts the ionospheric divergence under the assumption that the ionospheric vertical delay is same for all the time shifts. Thus, we need to analyze the temporal correlation property of the ionospheric error using the 24-h observables from the P057 site.

**Figure 4:**
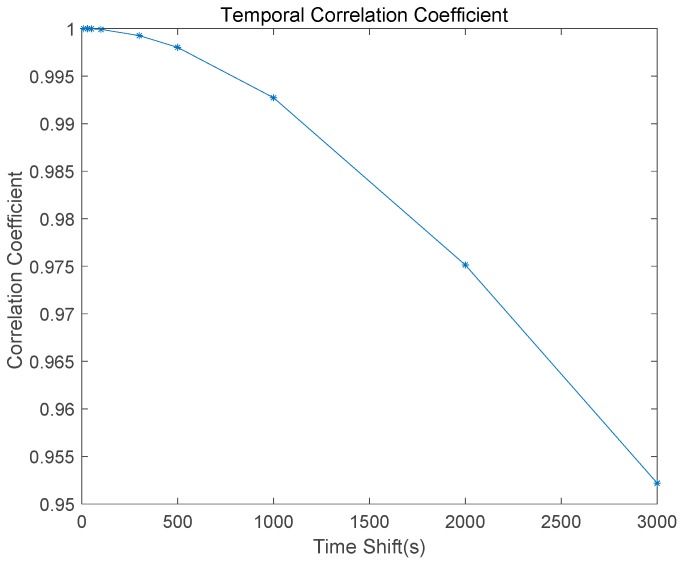
Temporal correlation coefficient of the ionospheric delay to various time shifts.

Once the ambiguity of the carrier phase has been determined during the cycle-slip free session, the ionospheric error can be obtained. We analyzed the temporal correlation of the calculated 24-h ionospheric error for all the satellites to various time shifts, 10, 30, 50, 100, 300, 500, 1000, and 2000 s. As shown in Figure 4, it is obvious that the larger the time shift, the smaller the temporal correlation coefficient of the ionospheric delay. While the coefficient is almost 1.00 for a time shift less than 100, the time interval of 3000 s reduces it to 0.95. It is important that the time constant of the Hatch filter in GBAS (ground based augmentation system) Approach Service Type C (GAST C) is 100 s and 30 s for GAST D [19]. 

**Figure 5:**
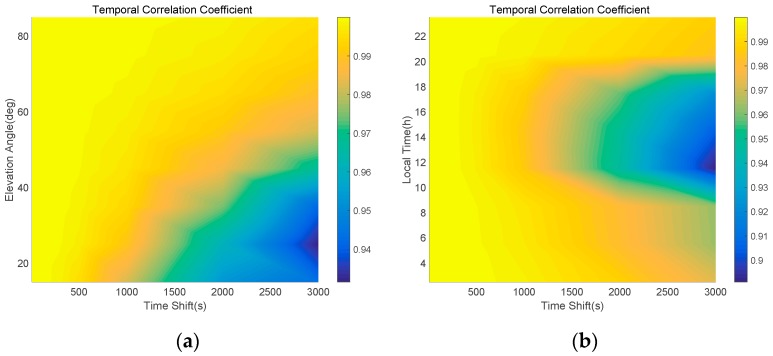
Temporal correlation coefficient of the ionospheric delay due to various time shifts, elevation angles (**a**), and local time (**b**).

Generally, the variation of the ionospheric delay for low-elevation satellites in the afternoon is relatively high; thus, we further study the effect of the elevation angle and local time on the ionospheric temporal coefficient. As expected, Figure 5 shows that the temporal correlation of high-elevation satellites during the night is strong. The coefficient for the elevation angle at 15° with a time constant of 100 s is 0.9998, which is the same as the value of the elevation angle at 85° with a time constant of 1000 s. The temporal correlation coefficient with 100 s time shift is also 0.9998 at 2 p.m., which can be obtained when the time constant is increased to 300 s at 10 p.m.

Since temporal correlation properties are similar for all elevation angles and local times, when the smoothing window width is assigned narrower than 100 s, the fixed small smoothing constant of the typical Hatch filter prevents the undesired divergence due to the highly correlated ionospheric error instead of the noise reduction. On the other hand, a fixed wide smoothing window of the SF divergence-free Hatch filter may put an overweight on the less-correlated ionospheric variation; the assumption of $\delta {\dot{I}}_{v}\;=0$ in Equation (25) during the day is not entirely valid as during the night. Therefore, the adaptive smoothing constant described in Equation (35) is effective in reducing the code noise when ionospheric activity is not active and in preventing the filter divergence due to the less-correlated ionospheric error during the afternoon. 

#### 4.2.3. Ionospheric Error Rate Estimation by SBAS Correction

**Figure 6:**
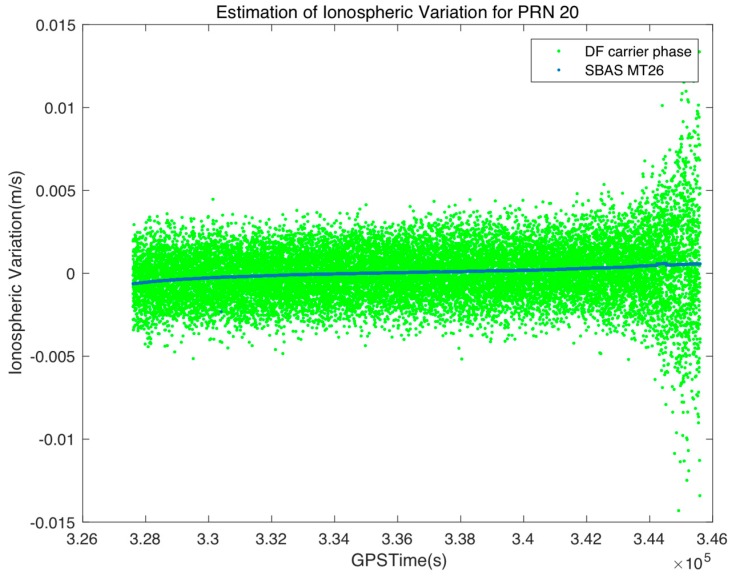
Result of the ionospheric error rate estimated by the dual-frequency carrier phase and SBAS MT26.

The most important assumption of the proposed SF divergence-free Hatch filter is that the rate of ionospheric error estimated by SBAS MT26 provides sufficiently good performance that does not degrade the filter performance. Figure 6 shows the estimation result with the combination of SBAS MT26 and DF carrier phase for the measurement of PRN 20. Based on the result that the rate calculated by MT26 passes through the center of the estimation by the DF measurement, we can verify that the estimator for the SF measurement and SBAS works well. There was no serious discontinuity in the estimates; therefore, the filter performance seems to be well maintained after the replacement of the DF into the SF.

It is interesting to note here that the noise of the estimation by DF is much larger than that by SF. From Equation (14), the estimation of the ionospheric variation by the DF measurements include error due to the carrier-phase noise, as expressed by Equation (37), while Equation (25) includes no noise-related term:

(37) $$\sigma_{\Delta {\hat{I}}_{DF}}^{2}=\frac{1}{{\left(\gamma -1\right)}^{2}}\left(\sigma_{\Delta \phi_{L1}}^{2}+\sigma_{\Delta \phi_{L2}}^{2}\right)$$

**Figure 7:**
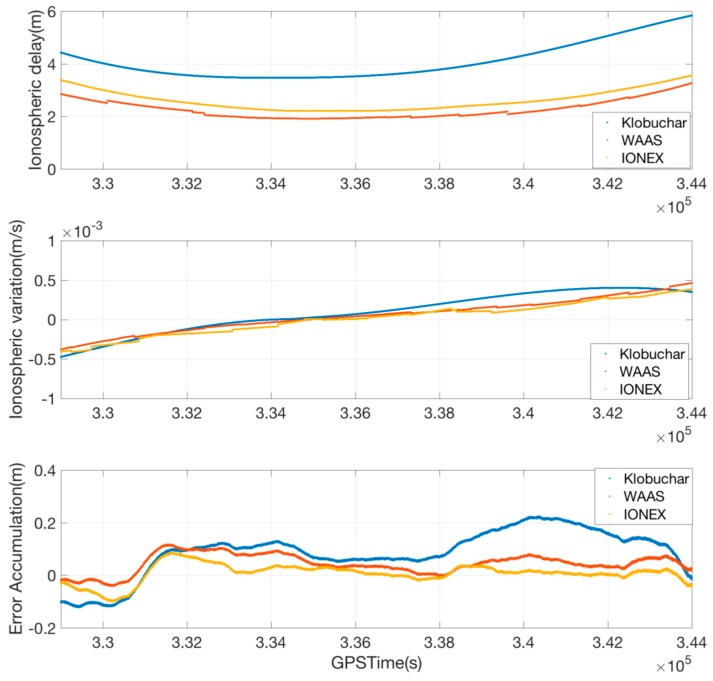
Performance comparison of the ionospheric variation modeling for PRN 20 at P057 site.

IONEX (ionospheric exchange) data and the Klobuchar model can be used to calculate the rate of the ionospheric delay, and we need to prove that the SBAS MT 26 provides better performance or a more practical method than these alternatives, the Klobuchar model and the IONEX data. IONEX data containing vertical total electron content (TEC) is provided by the IGS (international GNSS service) global network with a latency of 24 h to 11 days. The estimation performance of the IONEX is excellent enough to be usually used as a true reference of the global TEC, but it cannot be used in a real-time process because of the latency. Several service providers offer real-time TEC data in a suitable format such as SSR (state space representative), but an additional data channel is required to implement it for the local DGNSS or SBAS DGPS. In this sense, IONEX data is not a practical option to be used in the legacy DGNSS despite its superior performance. On the other hand, the Klobuchar model is an effective approach to mitigate the ionospheric delay for GPS single-frequency users [20]; however, it cannot follow the real-time ionospheric variation as shown in the upper graph of Figure 7 because it introduces a simplified model and many geometric approximations represented by four pre-defined broadcast coefficients. Although the estimation of the ionospheric delay rate by the broadcast model looks relatively better (middle of the Figure 7) than that of the ionospheric delay, the errors accumulated for the smoothing time by the Klobuchar model is larger than those by the SBAS MT 26. According to Equation (35), the time constant calculated by our suggested algorithm is up to 1000 s or more, thus the residual errors of the suggested SF divergence-free Hatch filter need to be compared to those of other methods after being accumulated for the last 1000 epochs. The RMS of the accumulated ionospheric residual errors, the 0.26 m, which is far larger than 0.10 m of the SBAS MT 26 and 0.06 m of the IONEX. Therefore, the estimation of the ionospheric variation by the SBAS MT 26 is a practical and accurate solution for the divergence compensation of the SF Hatch filter.

### 4.3. Performance Analysis in Range Domain

To compare the general characteristics of the conventional Hatch filter, DF divergence-free Hatch filter, and the suggested SF divergence-free Hatch filter, we applied three kinds of filters to the P057 data for various smoothing window widths of 25, 50, 100, 300, 500, and 1000, and then analyzed the performance of each filter in a range domain. To evaluate the filter performance, we calculated the residual error by the difference between the filtered pseudo-range and the true observable, which had been obtained by post-processing the carrier phase.

**Figure 8:**
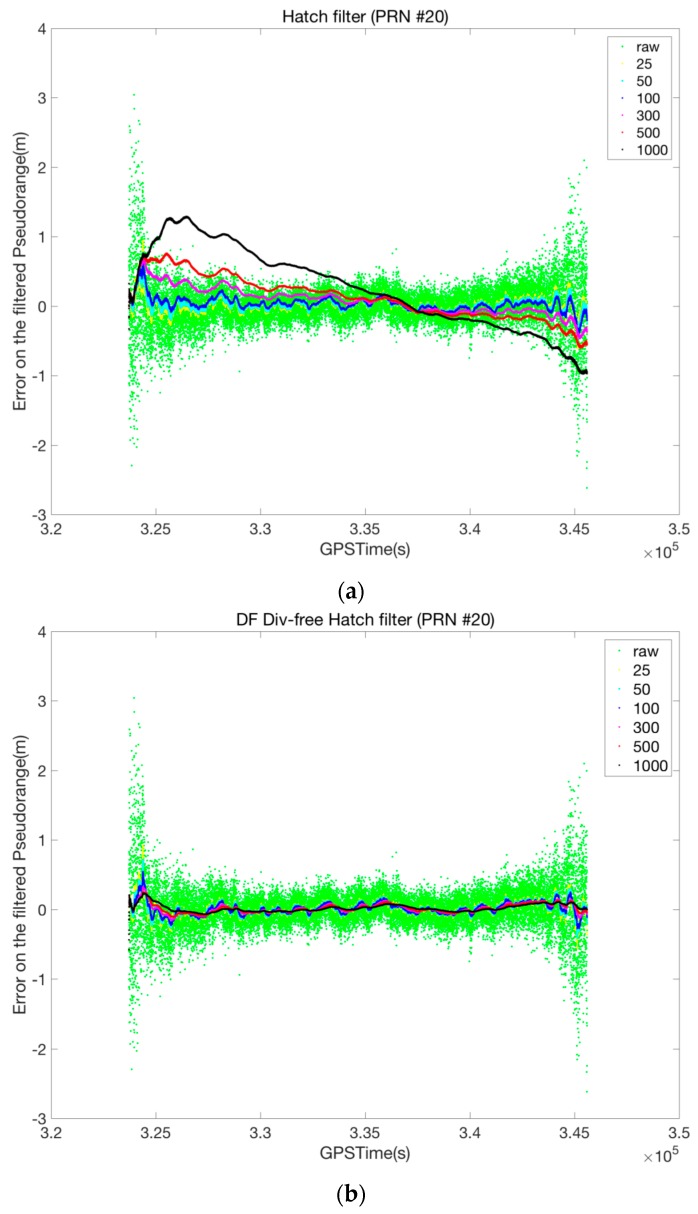

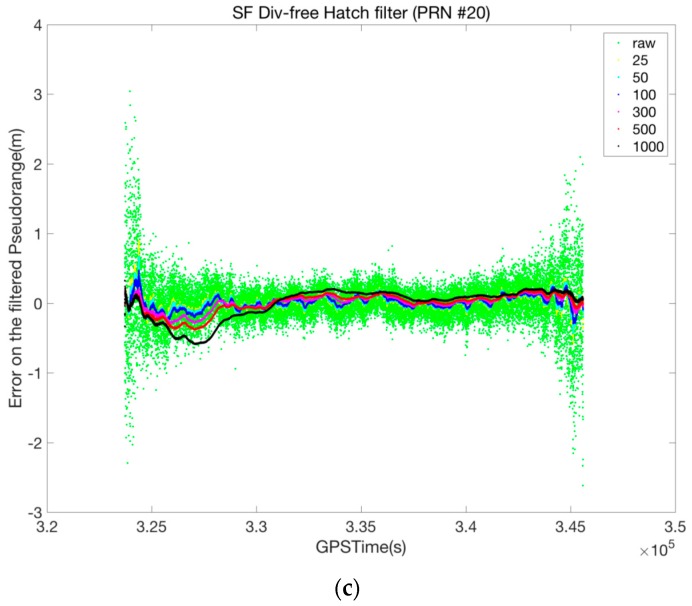
Smoothing filter performance for various smoothing window widths (**a**) Hatch filter; (**b**) DF divergence-free Hatch filter; (**c**) SF divergence-free Hatch filter.

Figure 8 shows the smoothing performances of each filter for the PRN 12 satellite, the elevation of which varies from 5° to 86°. In the case of the conventional Hatch filter, a smoothing window of width greater than 100 reduced the noise level but caused a large divergence error, while the DF divergence-free Hatch filter, even with a smoothing window width of 1000, was effective for noise reduction without a significant ionospheric-induced divergence. On the other hand, the SF divergence-free Hatch filter can reduce the measurement noise without a severe bias even when the smoothing time constant is as large as 1000. 

**Figure 9:**
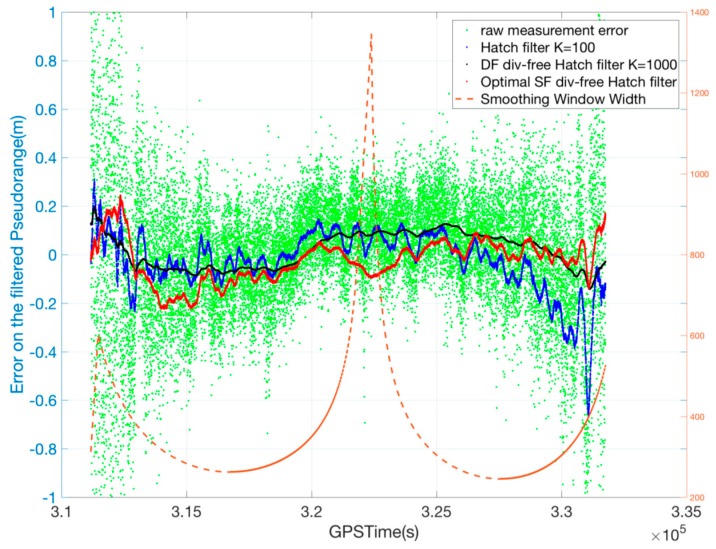
Performance of SF divergence-free Hatch filter for PRN 12.

Figure 9 describes the variation of the optimal smoothing constant and the resulting residual error of the optimal SF divergence-free Hatch filter, and the results of the conventional Hatch filter (K = 100) and DF divergence-free Hatch filter (K = 1000) are shown for reference. The averaging constant varies from 245 to 1345 as the elevation angle changes. Throughout the session, the residual error of the optimal SF divergence-free Hatch filter is well bound within 0.2 m, which is not as small as the value of 0.15 of the DF divergence-free Hatch filter but better than that of the conventional Hatch filter. In particular, the error of the optimal SF divergence-free Hatch filter at a GPSTime of 325,717 s is 0.01 m, while that of the conventional Hatch filter is −0.67 m. 

**Figure 10:**
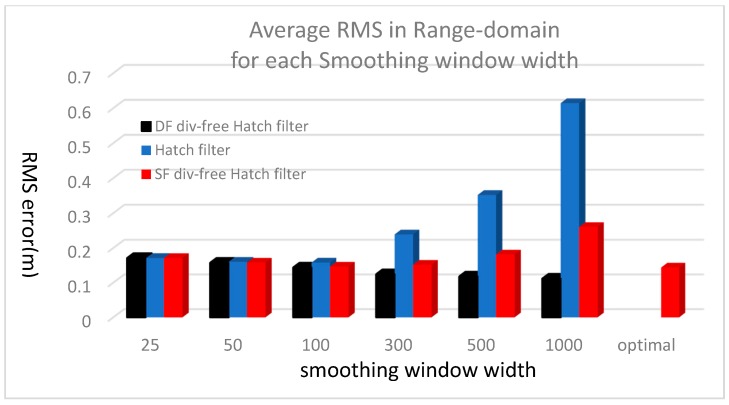
Average RMS in range-domain for each smoothing window width.

The range-domain error analysis results for all the satellites are summarized in Figure 10 and Table 2. For 25-s averaging, the performances of all the filters are almost the same. The RMS error of the DF divergence-free Hatch filter decreases as its smoothing constant increases, while that of the conventional Hatch filter increases sharply, especially when the width is greater than 100. The error of the SF divergence-free Hatch filter also increases if the averaging constant is greater than 300; however, the rate of increase is much less than that of the conventional Hatch filter.

Figure 10 and Table 2 also compares the performance of the optimal SF divergence-free Hatch filter with those of the filters with a fixed smoothing window. As described above, the smoothing window width can be as large as 1000 or more depending on the elevation angle, but its RMS error in the range domain remains at the smallest level among all fixed-width trials. 

### 4.4. Performance Analysis in Position Domain

From the previous results, we determined that the best performance can be obtained when the smoothing constants of the conventional, DF divergence-free, and SF divergence-free Hatch filter are 100, 1000, and 300, respectively. Thus, we computed the SBAS positions after setting the smoothing window width for each filter technique as the value yielding the best performance, and then compared the result with that obtained from the optimal SF divergence-free Hatch filter.

**Figure 11:**
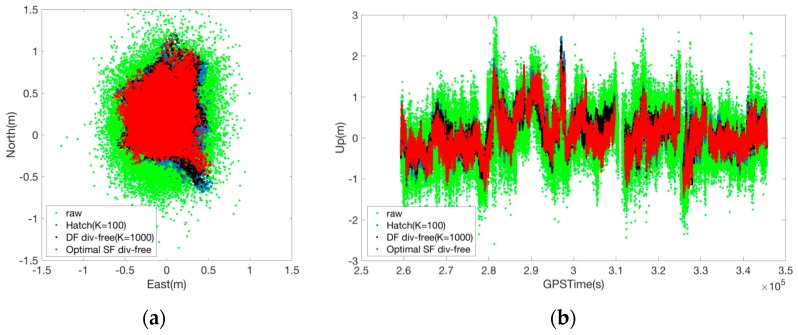
Error distribution for various smoothing filters. (**a**) horizontal; (**b**) vertical.

As is evident from Figure 11 and Table 3, the optimal SF divergence-free Hatch filter can reduce the RMS error of the conventional Hatch filter by 2 cm (horizontally) and 4 cm (vertically). From the statistical point of view, the position accuracy is not significantly improved, and the suggested filter is effective in bounding error divergence during the daytime.

**Figure 12:**
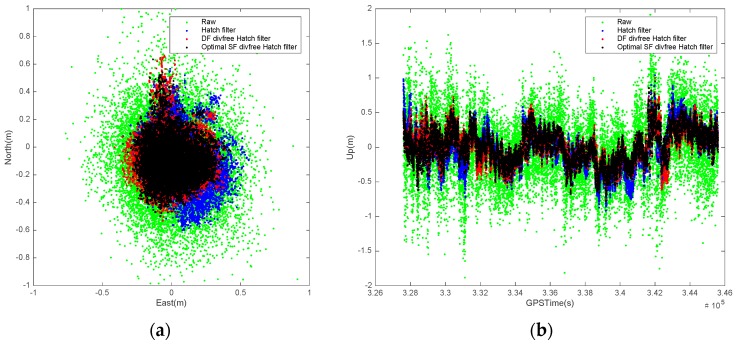
Filter performance comparison during daytime. (**a**) horizontal; (**b**) vertical.

Figure 12 zooms in the Figure 11 and shows the results for the daytime. Ionospheric activity is high during the afternoon (12:00–17:00), thus, ionosphere-induced error included in the Hatch filter is clearly visible. The SBAS position errors of the SF and DF divergence-free Hatch filters are normally distributed relative to those of the conventional Hatch filter, while the residual errors of the Hatch filter are located in the southeast direction. In particular for 329,011~331,235 s and for 339,151~345,052 s, the biggest error of the conventional Hatch filter is 57 cm and is biased, however, the DF divergence-free Hatch filter and the suggested algorithm bind the errors within 37 cm and do not cause a noticeable ionosphere-induced error in the horizontal plane.

**Figure 13:**
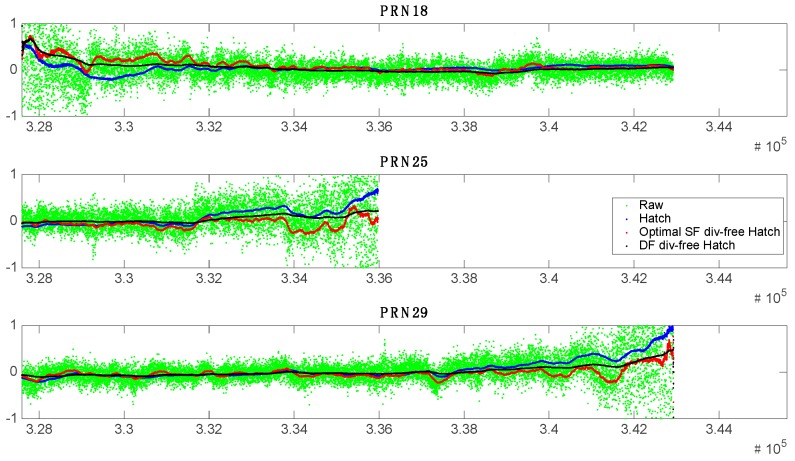
Residual error in range-domain for PRN #18, 25, and 29.

The residual error variations of several satellites during these periods are shown in Figure 13. All the filters can smooth the measurements for the highly-elevated satellites sufficiently, thus, their residual errors are almost zero for the high elevation angle cases having negligible temporal ionospheric delay gradients. Each filter, however, shows different tendency to each other for the measurements from low elevation satellites, often having large temporal ionospheric delay gradients. The DF divergence-free Hatch filter does not allow a large residual error even when the PRN 25 and 29 satellite is almost setting down. Although the low elevation satellite errors become fluctuated after smoothed by the SF divergence-free Hatch filter, the errors are still near to zero and placed on the overall tendency of that of DF divergence-free Hatch filter. On the other hand, the residual errors of the conventional Hatch filter grow up slowly as the elevation of the satellite decreases. Its errors for the new satellite (PRN 18) also gradually increases after the filter initialization, while the errors of other filters stay near to zero, with only a slight fluctuation after a convergence.

**Figure 14:**
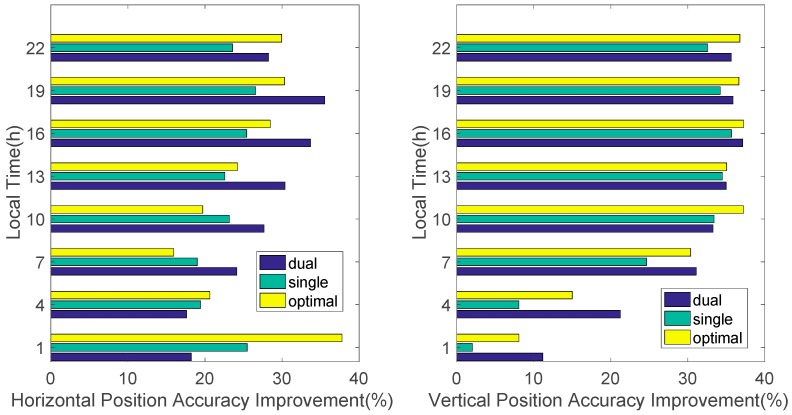
Position accuracy (RMS) improvement by local time.

We also analyzed the effect on the position domain due to the variation in the temporal correlation of the ionospheric error throughout the day. For analysis, the accuracy improvement, by applying each filter to the raw GNSS code measurement in local time, was calculated. As shown in Figure 14, the performance of the optimal SF divergence-free Hatch filter is superior to that of the Hatch filter for most of the day. The typical Hatch filter provides a comparable or slightly better performance than the suggested algorithm only just before noon, when ionospheric activity becomes more active so that $\delta {\dot{I}}_{v}$ is a little larger to be assumed as 0 for the assigned smoothing time constant. However, the suggested adaptive smoothing window contributes to a larger accuracy improvement throughout the day and it even provides better performance than the DF filter at night. 

The RMS value of the Hatch filter horizontal position error is 26 cm during the daytime (12:00~18:00), while those of the DF and optimal SF filters are 21 cm and 22 cm. The vertical error RMS of 32 cm also has been reduced to 24 cm and 28 cm by replacing the Hatch filter into DF and optimal SF Hatch filter as summarized in Table 4. Ninety-five percent of the errors have been distributed horizontally by 28 cm and vertically by 36 cm from the true position by the suggested algorithm, which were over 31 cm and 42 cm apart when smoothed by the Hatch filter. The accuracy improvement of the 95% error distribution is 34% and 42%, which are comparable to the DF filter.

**Figure 15:**
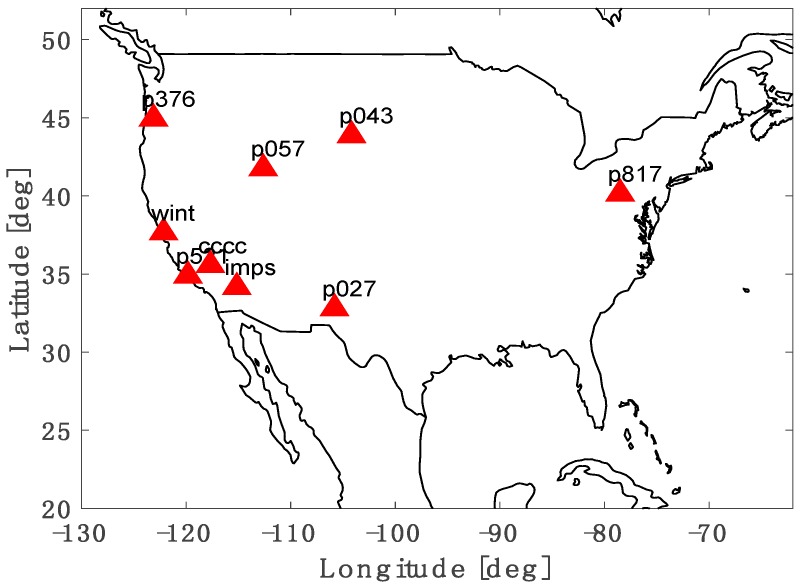
GNSS reference site distribution.

Lastly, we selected nine stations that can provide GNSS observables every second as shown in Figure 15 in order to analyze the spatial effect of the site on the position accuracy and to verify the generic applicability of the suggested algorithm. We collected the code and carrier measurements of each site from 13:00 to 15:00 local time, which includes the time of the strongest ionospheric activity, 14:00. The Hatch filter with a fixed time constant of 100 s and the optimal SF divergence-free Hatch filter were applied to the 2-h data. 

As summarized in Table 5, the overall performance has been improved by adjusting the ionospheric divergence and applying the adaptive smoothing window to the typical filter. The horizontal and vertical error RMS decreased by 2.5~5.3 cm and 0.8~11.7 cm, respectively, and the 95% error was also reduced by up to 15.3 cm and 18.8 cm. Therefore, we conclude that the proposed optimal SF divergence-free Hatch filter can improve the accuracy of the filter, as well as bind the divergence error.

## 5. Conclusions

The Hatch filter is one of the most commonly used smoothing filters, and it can reduce the pseudo-range noise of an SF receiver very effectively. Due to the simplicity of constructing a Hatch filter and its effectiveness in reducing the measurement noise, it is more widely used than other sophisticated filters, such as the DF divergence-free Hatch filter and Kalman filter. The performance of the Hatch filter is profoundly coupled to the averaging constant, and previous studies had focused on minimizing the divergence-induced error. 

In this paper, we proposed the use of the ionospheric correction message of SBAS in order to reduce the ionospheric-induced divergence of the conventional Hatch filter, and we showed how to calculate the optimal smoothing constant to minimize the residual error. By analyzing 24-h measurement in the range and position domain, we validated the proposed algorithm and its effectiveness to reduce noise without severe divergence. The error statistics of the proposed optimal SF divergence-free Hatch filter is found to be similar to those of the DF divergence-free Hatch filter. Moreover, the proposed adaptive smoothing window can prevent users from experiencing a severe bias due to a steep ionospheric delay variation for a low elevation satellite during daytime; it can reduce the 32 cm vertical error RMS of the conventional Hatch filter by 8 cm, vertical accuracy improvement of 25%. It can also bind the 95% error within 27 cm horizontally, and 35 cm vertically, which were 32 cm and 42 cm by the Hatch filter. 

Considering that SF receivers dominate the GNSS market and that most of these receivers include the SBAS function, the filter suggested in this paper is very practical to improve the performance of most receivers while maintaining the recursive and simple form of the conventional Hatch filter. It is also of great value in that it can make the DGPS performance of the low-cost SF receivers comparable to that of DF receivers.

## Figures and Tables

**Table 1:** Estimation result of parameters of the CNMP model.

$x_{0}$	$x_{1}$	$x_{2}$
0.1420	1.6309	9.9000

**Table 2:** RMS of the residual error in the smoothed pseud-orange.

Filtering Method	25	50	100	300	500	1000
DF div-free	0.1713	0.1576	0.1440	0.1247	0.1182	0.1118
Hatch	0.1717	0.1603	0.1583	0.2394	0.3521	0.6147
SF div-free	0.1714	0.1579	0.1465	0.1518	0.1813	0.2604
Opt. SF div-free	0.1440					

**Table 3:** Statistics of the position error.

Filtering Method	Horizontal Error		Vertical Error	
	RMS (m)	Max (m)	RMS (m)	Max (m)
Raw	0.4229	1.7835	0.6102	3.6417
DF div-free (K = 1000)	0.3190	1.1072	0.4106	1.5481
Hatch (K = 100)	0.3316	1.0762	0.4497	1.7313
SF div-free (K = 300)	0.3123	1.0801	0.4121	1.7168
Opt. SF div-free	0.3146	1.0719	0.4111	1.7542

**Table 4:** Statistics of the position error during daytime (12:00–18:00).

Accuracy		Raw	DF Div-Free	Hatch	Optimal SF Div-Free
Horizontal Accuracy	RMS (m)	0.3440	0.2182	0.2592	0.2282
	95% (m)	0.4186	0.2635	0.3195	0.2764
	Improvement	-	37%	24%	34%
Vertical Accuracy	RMS (m)	0.4889	0.2847	0.3236	0.2413
	95% (m)	0.6089	0.3537	0.4200	0.3545
	Improvement	-	42%	31%	42%

**Table 5:** Statistics of the position error for various sites (13:00–15:00).

Site Name	Hatch Filter						Optimal SF Div-Free Hatch Filter					
	Horizontal			Vertical			Horizontal			Vertical		
	RMS	95%	MAX	RMS	95%	MAX	RMS	95%	MAX	RMS	95%	MAX
p376	0.542	0.838	1.166	0.492	0.904	1.193	0.493	0.700	0.875	0.455	0.804	1.112
p043	0.404	0.701	0.907	0.761	1.356	1.758	0.363	0.606	0.778	0.687	1.245	1.531
p057	0.319	0.548	0.777	0.782	1.396	1.731	0.266	0.448	0.668	0.734	1.332	1.562
p817	0.536	0.809	0.977	0.723	1.219	3.006	0.499	0.761	0.984	0.697	1.183	2.978
wint	0.726	1.138	1.382	0.715	1.299	1.939	0.697	0.985	1.221	0.719	1.267	1.964
cccc	0.461	0.879	1.130	0.287	0.589	0.897	0.427	0.771	0.976	0.3	0.573	0.874
p521	0.663	1.126	1.449	0.373	0.741	1.095	0.627	1.006	1.266	0.365	0.656	0.889
imps	0.415	0.742	1.318	0.452	0.961	1.820	0.391	0.650	1.273	0.444	0.949	1.827
p027	0.360	0.669	0.906	0.333	0.626	1.049	0.327	0.545	0.744	0.216	0.438	0.755
